# Distal organ inflammation and injury after resuscitative endovascular balloon occlusion of the aorta in a porcine model of severe hemorrhagic shock

**DOI:** 10.1371/journal.pone.0242450

**Published:** 2020-11-17

**Authors:** Yansong Li, Michael A. Dubick, Zhangsheng Yang, Johnny L. Barr, Brandon J. Gremmer, Michael L. Lucas, Corina Necsoiu, Bryan S. Jordan, Andriy I. Batchinsky, Leopoldo C. Cancio

**Affiliations:** 1 Department of Expeditionary Critical Care Research, US Army Institute of Surgical Research, Fort Sam Houston, Texas, United States of America; 2 Department of Damage Control Resuscitation, US Army Institute of Surgical Research, Fort Sam Houston, Texas, United States of America; 3 U. S. Army Burn Center, US Army Institute of Surgical Research, Fort Sam Houston, Texas, United States of America; Leiden University Medical Center, NETHERLANDS

## Abstract

**Background and objective:**

Resuscitative Endovascular Balloon Occlusion of Aorta (REBOA) has emerged as a potential life-saving maneuver for the management of non-compressible torso hemorrhage in trauma patients. Complete REBOA (cREBOA) is inherently associated with the burden of ischemia reperfusion injury (IRI) and organ dysfunction. However, the distal organ inflammation and its association with organ injury have been little investigated. This study was conducted to assess these adverse effects of cREBOA following massive hemorrhage in swine.

**Methods:**

Spontaneously breathing and consciously sedated Sinclair pigs were subjected to exponential hemorrhage of 65% total blood volume over 60 minutes. Animals were randomized into 3 groups (n = 7): (1) Positive control (PC) received immediate transfusion of shed blood after hemorrhage, (2) 30min-cREBOA (A30) received Zone 1 cREBOA for 30 minutes, and (3) 60min-cREBOA (A60) given Zone 1 cREBOA for 60 minutes. The A30 and A60 groups were followed by resuscitation with shed blood post-cREBOA and observed for 4h. Metabolic and hemodynamic effects, coagulation parameters, inflammatory and end organ consequences were monitored and assessed.

**Results:**

Compared with 30min-cREBOA, 60min-cREBOA resulted in (1) increased IL-6, TNF-α, and IL-1β in distal organs (kidney, jejunum, and liver) (*p* < 0.05) and decreased reduced glutathione in kidney and liver (*p* < 0.05), (2) leukopenia, neutropenia, and coagulopathy (*p* < 0.05), (3) blood pressure decline (*p <* 0.05), (4) metabolic acidosis and hyperkalemia (*p* < 0.05), and (5) histological injury of kidney and jejunum (*p* < 0.05) as well as higher levels of creatinine, AST, and ALT (*p* < 0.05).

**Conclusion:**

30min-cREBOA seems to be a feasible and effective adjunct in supporting central perfusion during severe hemorrhage. However, prolonged cREBOA (60min) adverse effects such as distal organ inflammation and injury must be taken into serious consideration.

## Introduction

Major torso injuries in recent wars underlined the difficulty in controlling non-compressible torso hemorrhage (NCTH) [1–3]. Deaths because of exsanguination secondary to injuries involving truncal, abdominal and pelvic vessels are considered as potentially survivable [4]. Traditionally, resuscitative thoracotomy or laparotomy are used in an effort to prevent cardiovascular collapse by increasing afterload and central aortic pressure until hemostasis may be achieved [5, 6]. These invasive steps are often undertaken after loss of pulses. Less invasive endovascular techniques such as balloon occlusion of the aorta, is an alternative to open surgery [3, 7–13]. We have shown that complete resuscitative endovascular balloon occlusion of the aorta (cREBOA), resulted in the restoration of carotid flow (Q_carotid_ “cerebrovascular resuscitation”) as effectively as immediate transfusion of shed blood, with equivalent 4-hour survival [14].

The occlusion of the thoracic aorta is known to restore central aortic pressure in hemorrhagic shock [10, 14–16] and has been shown in an animal model of uncontrolled hemorrhage to increase survival in comparison with resuscitation using normal saline without hemorrhage control [8]. Deleterious effects of aortic occlusion and the magnitude of these effects are related to duration of occlusion [9, 15, 17–20]. Although the outcome of cREBOA is the same as cross clamping of the aorta through a thoracotomy, the physiologic insult by the endovascular approach has been shown to be less than with the open method [7]. However, there is a finite range of time for which the aortic balloon occlusion might be applied in one setting. A non-lethal, 35% hemorrhage in swine revealed up to 90 min as the limit of use before significant and potentially irreversible ischemia-induced organ injury occurs [9]. More severe hemorrhage (e.g, 60%), known to be fatal without resuscitation [21], is likely near the limit of physiologic tolerance for these mini-swine. Although aortic balloon occlusion restored central aortic pressure to near pre-hemorrhage values [9, 22], endpoints of resuscitation with a decisively spared circulatory volume provided by aortic occlusion are relatively unknown. We hypothesized that prolonged cREBOA (60 min) would cause distal organ inflammation and injury. Based on these premises, this study explored the effects of 30min- and 60min-cREBOA (two natural testable thresholds of cREBOA duration in human clinical use) at aortic zone 1 in a porcine model of severe hemorrhagic shock (65% total blood volume) on distal organ inflammation and injury.

## Materials and methods

Research was conducted in compliance with the Animal Welfare Act, the implementing Animal Welfare regulations, and the principles of the Guide for the Care and Use of Laboratory Animals, National Research Council. The facility’s Institutional Animal Care and Use Committee approved all research conducted in this study. The facility where this research was conducted is fully accredited by the AAALAC.

### Experimental design

**Fig 1:**
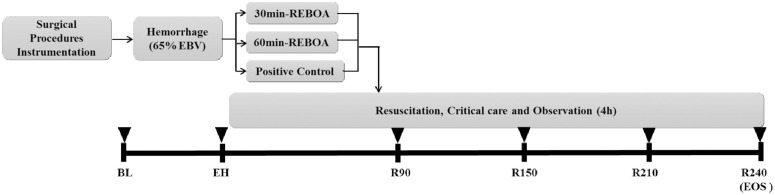
Overview of experimental design. BL, baseline; EBV, estimated blood volume; EH, end of hemorrhage; EOS, end of study; R90, R150, R210 and R240 are 90-, 150-, 210-, and 240-min post-resuscitation, respectively; cREBOA, complete resuscitative endovascular balloon occlusion of the aorta.

Sexually mature, non-castrated male Sinclair Miniature Swine (Sinclair Bio-Resources, Columbia, MO), 38 ± 9 kg, were used in this study. Animals were specially bred, socialized, vaccinated, and free from common domestic swine diseases. Total of 21 animals were randomized into three subgroups, n = 7 each. All animals underwent 65% total blood volume hemorrhage, with subsequent care as follows: 1) Positive control (PC): 65% blood volume hemorrhage and then immediate transfusion of shed blood. 2) A30: cREBOA for 30 minutes, followed by transfusion of shed blood just prior to balloon deflation. 3) A60: cREBOA for 60 minutes, followed by transfusion of shed blood after balloon deflation as described [14] (Fig 1).

### Surgical procedures and cREBOA insertion

All surgical procedures were described previously [14]. In brief, after an overnight fast, with water available *ad libitum*, animals were transferred to a procedure room where they were received buprenorphine hydrochloride (0.05 mg/kg IM) as preemptive analgesia and anesthetized with Telazol^®^ (Zoetis, Florham Park, NJ, 4mg/kg IM). Animals were endotracheally intubated, and inhalational anesthesia was maintained using isoflurane (1–5 vol %), delivered in 100% oxygen. A Dräger Fabius GS anesthesia machine (Dräger Medical In., Telford, PA) was used. Vascular cannulation was performed as follows. In the left neck, an 8.5 Fr introducer sheath (Teleflex, Inc., Wayne, PA) was inserted into the external jugular vein via a cut-down. An Arrow 7 Fr multilumen central venous catheter (Arrow International Inc., Reading, PA) was inserted via the introducer sheath. A 6 Fr introducer sheath (Teleflex, Inc.) was inserted into the common carotid artery, and a 5 Fr VolumeView catheter (Edwards Lifesciences Corp., Irvine, CA) was then placed. This catheter was connected via a high pressure monitoring line (Smith Medical ASD Inc., Dublin, OH) to an Infinity HemoMed Pod (Dräger Medical Inc.). In the left neck, an 8.5 Fr introducer sheath (Teleflex, Inc., Wayne, PA) was inserted into the external jugular vein via a cut-down. An Arrow 7 Fr multilumen central venous catheter (Arrow International Inc., Reading, PA) was inserted via the introducer sheath. A 6 Fr introducer sheath (Teleflex, Inc.) was inserted into the common carotid artery, and a 5 Fr VolumeView catheter (Edwards Lifesciences Corp., Irvine, CA) was then placed. This catheter was connected via a high pressure monitoring line (Smith Medical ASD Inc., Dublin, OH) to an Infinity HemoMed Pod (Dräger Medical Inc.). In the right groin, the common femoral artery was accessed via a cut-down and cannulated with a 7 Fr introducer sheath (Teleflex Inc.), for future cREBOA insertion (discussed later). In the left groin, the common femoral artery was accessed via a cut-down and cannulated with an 8 Fr introducer sheath (Teleflex Inc.), and a high-pressure tubing (ICU Medical) was advanced through the sheath. A tracheostomy was performed, and a 10-mm cuffed endotracheal tube (Lo-Pro, Mallinckrodt Inc., St. Louis, MO) was inserted.

After instrumentation, all incisions were closed with sutures or staples, and the wounds were infiltrated with 0.5% bupivacaine. Animals were transferred into a custom-built sling, which allows an animal to remain in an anatomic quadruped position. Inhalational anesthesia was discontinued, and buprenorphine continuous intravenous infusion was started at 5 mL/h (1 Kg/kg/h). Once the animal recovered sufficiently to breathe spontaneously, a midazolam bolus (2 mg/kg) was administered intravenously, and a continuous intravenous infusion of 0.1 mg/kg per hour was started. The infusion rates were decreased by half when mean arterial pressure (MAP) decreased below 80 mm Hg and were held when MAP was lower than 50 mm Hg. Analgesia was titrated to elimination of pain or distress as evident by physiological parameters (blood pressure and heart rate changes). Anesthetic depth was monitored throughout all procedures via bispectral index (to maintain a value of 80–90).

### Vital sign monitoring

Vital function parameters including heart rate and arterial blood pressure were also periodically monitored via ta high pressure monitoring line (Smith Medical ASD Inc., Dublin, OH) connected to the left common carotid artery catheter, and recorded and stored using proprietary data acquisition software (Integrated Data Exchange and Archival [IDEA] system). Arterial pO_2_ and pCO_2_ Arterial blood gas analysis was performed at the bedside using an iSTAT 300-G blood analyzer (Abbott Point of Care Inc., Princeton, NJ; VetScan CG4+ and CG8+ cartridges, Abaxis Inc., Union City, CA).

### Hemorrhage shock and transfusion

Hemorrhage was performed through the left common femoral artery catheter using a computer-controlled peristaltic pump (Masterflex, Cole-Parmer, Vernon Hills, IL) as described [14]. Blood was removed via Tygon tubing (E-Lab [E3603] L/S 16, Cole-Parmer) into 500-mL Teruflex blood-collection bags containing citrate-phosphate-dextrose (CPD) with OPTISOL red/cell preservative solution (Terumo Corp., Tokyo, Japan). Estimated total blood volume (EBV) was calculated using the following formula: EBV = weight in kilogram × 65 mL/kg. After correcting for the blood volume withdrawn for baseline laboratory values (48.5 mL), animals were bled 65% of EBV over 60 minutes in an exponential manner [14]. Entirety of shed whole blood was returned at a rate of 100mL/min via the right external jugular vein using a computer-controlled peristaltic pump (Masterflex, Cole-Parmer, Vernon Hills, IL). IV calcium chloride was infused to reverse hypocalcemia induced by the citrated blood.

### REBOA device and insertion

REBOA (ER-REBOA, Pryor Medical, Inc.) was inserted at the end of hemorrhage via the 7 Fr introducer previously placed in the right common femoral artery. The intended zone of insertion for the balloon was between the left subclavian and the celiac arteries (zone 1). Insertion depth (40cn) was guided by the centimeter marks on the catheter.

### Measurement of circulating metabolic parameters

Blood samples were analyzed for pH, pCO_2_, pO_2_, HCO_3_, base excess/deficit (BE/BD), lactate, and glucose levels using i-STAT cartridges (Abbott Laboratories, Abbott Park, IL).

### Complete blood count (CBC) and coagulation parameter assessment

CBC and coagulation parameters (PT, prothrombin time; INR, international normalized ratio; and PTT, partial thromboplastin time) were measured using an ABX Pentra 120 hematology analyzer (ABX Diagnostics, Inc., Irvine, CA) and Start-4 (Diagnostica Stago, Rue des Freres Chausson, FranceTAGO) respectively.

### Assessment of circulating end-organ damage markers

Creatinine, alanine aminotransferase (ALT), aspartate aminotransferase (AST) and myoglobin were measured from blood samples using Dimension Xpand Plus Integrated Chemistry System (Siemens, Holliston, Mass).

### Histological evaluation

Under a general anesthesia animal will be euthanized at the end of study with Fatal Plus at a dose of 10ml/10lb intravenously. Following euthanasia, tissue samples (Liver, jejunum, and kidney,) for histology were collected and fixed in neutral buffered 10% formalin for 24 hours, trimmed, embedded in paraffin, sectioned at 4 μm, and stained with hematoxylin and eosin (H&E). Histologic images were recorded with a 10× objective under a slide scanner (Axio Scan.Z1 v1.0, Zeiss, Germany). Histologic grading of injury was performed by a single pathologist blinded to the identity of the animal. For the liver, five characteristics were scored (vascular congestion, thrombosis, hepatic apoptosis, cellular degeneration, and inflammation). The severity of the injury (score 0 = normal histology, score 1 = slight, score 2 = mild, score 3 = moderate and score 4 = severe alterations). For the jejunum, each slide was scored according to the following scale: 0, normal villi; 1, villi with tip distortion; 2, villi lacking goblet cells and containing Guggenheim’s spaces; 3, villi with patch disruption of the epithelial cells: 4, villi with exposed but intact lamina propria and epithelial cell sloughing; 5, villi in which the lamina propria was exuding; and 6, hemorrhaged or denuded villi. For the kidney, each slide was scored according to this scale: 0, normal histology; 1, slight alteration (loss of brush border, mild hydropic degeneration, mild congestion); 2, mild (intensive hydropic degeneration, mild vacuolization, and interstitial edema); 3, moderate (nuclear condensation, intensive vacuolization, moderate interstitial edema); and 4, severe (necrotic/apoptotic cells, denudation/rupture of basement membrane). For each slide, Total Injury Score (TIS) was calculated as the sum of the severity and the extent of injury. The grades for changes were assigned according to the extent (score 0, 1, 2, 3 and 4 for an extent of 0%, <25%, 25–50%, 50–75%, and 75–100% respectively). For most organs, several characteristics were scored and the choice of the features was suited to a particular organ as described [14].

### Measurement of tissue cytokines and oxidative stress

Liver, jejunum, and kidney tissue specimens were collected, and homogenized in 50 mM potassium phosphate buffer, pH 7.4. The cytokines (TNF-α, IL-1β, and IL-6) were detected using the MSD multiplex system (Rockville, MD). The reduced glutathione concentrations in the tissue were measured as previously described [23]. Briefly, the tissue extract (20 μl) was incubated with 120 μl of fresh prepared 5,5′-dithio-bis(2-nitrobenzoic acid) (DTNB) and glutathione reductase (GR) solution (DTNB:GR mixture: 1:1) for 30 seconds at room temperature followed by adding 60 μl of β-NADPH, The micro-plate was immediately read the absorbance at 412 nm by the microplate reader (Molecular Devices, San Jose, CA). For internal control, the concentrations of total protein were determined with a commercial kit (Pierce, Rockford, IL).

### Statistical analysis

Using SAS Power 9.2 and a detectable difference of at least 82%, 7 animals per group is required to achieve a power of 0.8 using an alpha = 0.05. Given that prior studies have showed that survival in aortic balloon occlusion groups at 30 and 60 minutes of occlusion ranging from 87.5% to 100%, we believe that using 7 animals per group will provide adequate power to compare survival versus the negative control group. Statistical analyses were performed using GraphPad Prism 6.0 (GraphPad Software, Inc., San Diego, CA) and Excel ver. 14.0. Data were statistically analyzed by Mann-Whitney test for non-parametric and semi-quantitative data. Data were expressed as mean ± standard deviation or standard error as indicated and significance was accepted at *p* < 0.05.

## Results

### Baseline characterization and mortality

There was no significant difference in baseline (BL) measurements of hemodynamic and metabolic parameters among the PC, A30 and A60 groups, except that PO_2_ were lower in both groups of A30 and A60 (p<0.05, vs. PC) (Table 1). Four-hour survival for the PC, A30, and A60 groups was 71% (5/7), 100 (7/7), and 100% (7/7) respectively (p = 0.1156, log-rank test). The death of 2 animals in the PC group occurred at 122min and 108min respectively, and was from cardiac arrest after balloon deflation. These 2 animals were similar to their cohort in all respects.

**Table 1:** Hemodynamics and metabolic characteristics in swine.

Parameters	Groups	Timeline					
		BL	EH	R90	R150	R210	EOS
**MAP (mmHg)**	**PC**	123.0±21.4	30.7±13.8	104.3±35.6	118.4±18.6	115.0±21.4	101.5±45.7
	**A30**	118.4±20.7	45.0±9.0^#^	115.6±13.8	148.1±23.8^#^	158.3±20.8^#^	152.6±26.9^#^
	**A60**	113.7±6.9	44.1±10.4*	**85.1±22.1**†	86.7±17.7*†	**96.4±20.0**†	**96.6±18.5**†
**SI**	**PC**	0.6±0.1	3.5±1.0	0.8±0.3	0.6±0.1	0.7±0.0	1.2±1.4
	**A30**	0.5±0.1	2.9±0.6	0.9±0.3	0.6±0.2	0.5±0.2	0.6±0.3
	**A60**	0.6±0.1	2.9±0.6	1.2±0.3	1.3±0.5*†	1.2±0.3*†	**1.2±0.3**†
**HCO3 (mmol/L)**	**PC**	30.7±0.9	19.2±4.1	25.6±6.4	30.6±0.9	30.6±1.1	26.2±8.3
	**A30**	30.5±2.7	23.9±3.4	20.3±2.5^#^	24.3±2.9^#^	25.7±3.7^#^	25.5±4.0
	**A60**	30.8±1.8	22.4±5.3	11.6±2.4*†	14.1±2.9*†	14.7±2.5*†	**17.6±4.0**†
**PO2 (mmHg)**	**PC**	88.1±9.8	85.9±37.7	83.3±11.0	85.2±3.0	77.5±3.7	66.8±22.4
	**A30**	73.3±11.3^#^	97.3±7.4	87.4±5.3	92.6±4.0^#^	88.7±4.3^#^	85.3±9.3^#^
	**A60**	78.1±6.9*	98.9±18.9	106.4±9.1*†	105.0±7.6*†	110.6±15.7*†	100.4±10.1*†
**PCO2 (mmHg)**	**PC**	47.3±1.8	44.0±14.0	48.1±17.1	44.6±1.2	44.2±1.6	61.0±25.0
	**A30**	47.1±5.2	28.9±4.5	35.9±4.1^#^	39.7±3.1^#^	40.6±3.3	39.9±3.9^#^
	**A60**	48.0±2.3	31.9±10.9	31.0±4.7*	30.2±6.0*†	30.8±5.6*†	35.5±6.5*
**Hb (mmol/L)**	**PC**	22.0±1.6	19.2±4.5	n/a	n/a	n/a	24.3±3.2
	**A30**	22.0±1.5	21.7±1.5	23.4±3.7	24.3±2.9	25.0±3.2	25.0±3.0
	**A60**	22.0±1.1	20.2±1.6	**28.2±2.0**†	n/a	n/a	32.7±1.8*†
**Hct (%)**	**PC**	32.9±2.6	29.6±4.1	n/a	n/a	n/a	36.5±5.9
	**A30**	32.7±1.5	31.7±3.3	34.8±5.0	35.2±4.7	35.4±4.8	35.3±5.0
	**A60**	32.7±2.5	31.1±2.2	n/a	n/a	n/a	46.9±2.7*†
**Gluc (mmol/L)**	**PC**	7.0±1.4	21.1±8.8	n/a	n/a	n/a	13.9±16.1
	**A30**	6.5±0.8	11.8±2.5^#^	n/a	14.9±1.9	n/a	12.4±3.5
	**A60**	6.5±0.7	13.2±3.5	n/a	n/a	n/a	17.9±8.9
**K**^**+**^ **(mmol/L)**	**PC**	3.8±0.2	4.9±1.4	n/a	n/a	n/a	4.3±0.7
	**A30**	4.0±0.2	4.0±0.2^#^	n/a	4.5±0.7	n/a	4.2±0.4^#^
	**A60**	4.0±0.2	4.3±0.3	n/a	n/a	n/a	7.1±1.2*

Abbreviations: BL, baseline; EH, end of hemorrhage; EOS, end of study; MAP, mean arterial pressure; N/A, not available; R, post-resuscitation; SI, shock index. Data was shown as Mean ± SD;

^#^ *p*<0.05, A30 *vs*. PC;

* *p*<0.05, A60 *vs*. PC;

^†^ *p*<0.05, A60 *vs*. A30. Data were analyzed by Mann-Whitney test.

### Effect of cREBOA duration on circulating metabolism and hemodynamics

**Fig 2:**
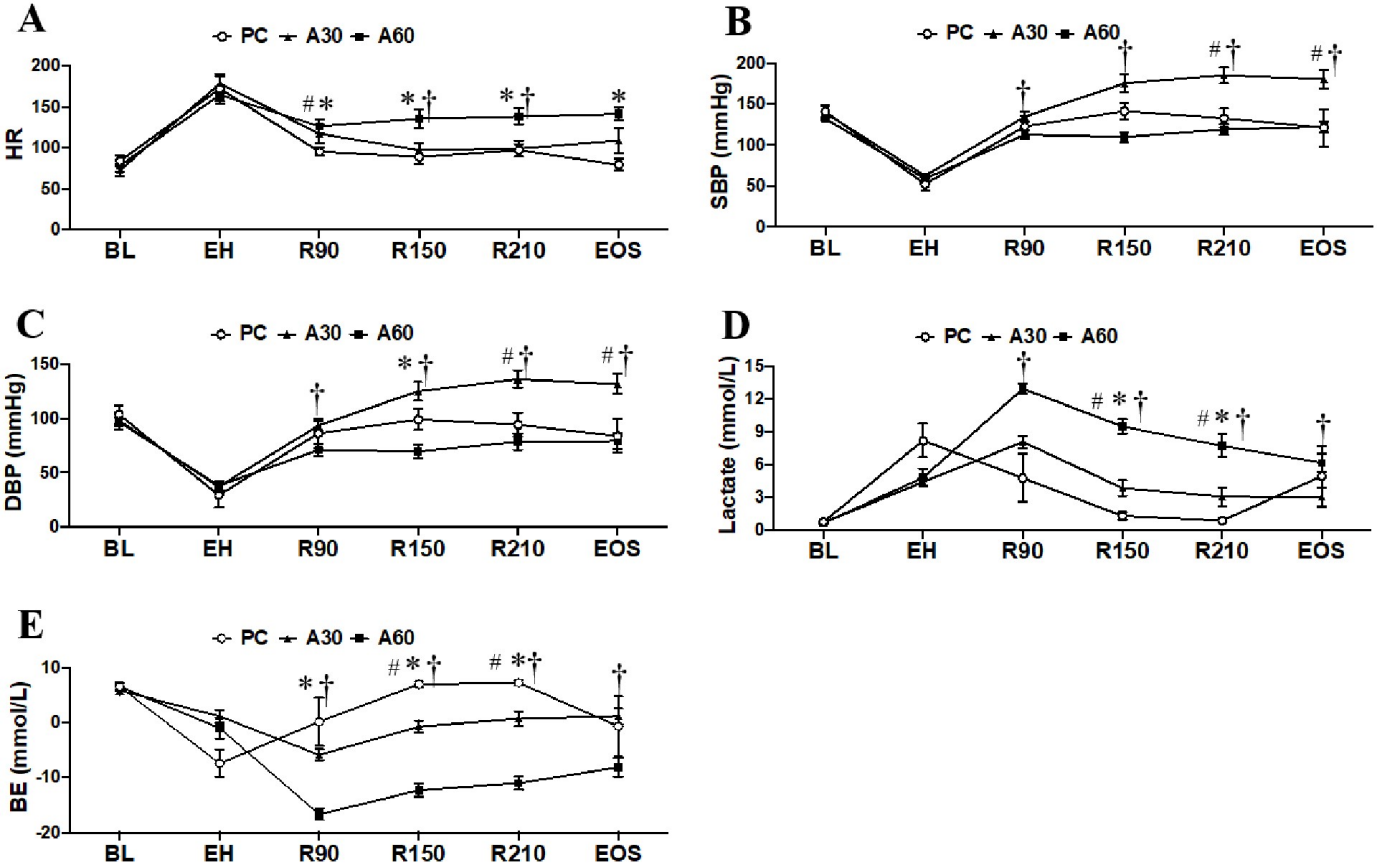
The hemodynamic and metabolic performance of swine undergoing cREBOA. Data are presented as mean ± SEM and were statistically analyzed using two-tailed Mann-Whitney test. ^#^ *p*<0.05, A30 *vs*. PC group; * *p*<0.05, PC *vs*. A60 group; † *p*<0.05, A60 *vs*. A30 group. A30, cREBOA for 30 minutes; A60, cREBOA for 60 minutes; DBP, diastolic blood pressure; PC, positive control. BE, base excess; EOS, end of study; HR, heart rate; SBP, systolic blood pressure.

As shown in Table 1 and Fig 2, all groups responded to resuscitation with a substantial improvement in blood pressure (SBP, systolic blood pressure; DBP, diastolic blood pressure; and MAP), heart rate (HR), and shock index (SI) throughout the resuscitation period. A30 group, but not A60 group, responded to cREBOA with a significant rise in blood pressure above the BL values from R90 to EOS (Table 1 and Fig 2, *p <* 0.05). The vital signs (SBP, DBP and MAP) of individual animals were shown in S1 Table. Compared to PC and A30 groups, A60 exhibited a higher HR and SI, which were most significant at R150, R210 and EOS after resuscitation (Fig 2A and Table 1, *p <* 0.05). However, lactate and BE concentrations in A60 group were significantly higher and lower respectively from R90 to EOS post-cREBOA indicating severe metabolic acidosis (Fig 2C and 2D, *p <* 0.05). Hemodynamic parameters in A60 such as SBP, DBP, and MAP were significantly lower than A30 values (*p <* 0.05) while hemoglobin (Hb), hematocrit (Hct), and blood potassium (K^+^) were significantly higher than both A30 and PC values (Fig 2B and 2C, and Table 1, *p <* 0.05). There was a trend toward greater levels of blood glucose at the EOS in A60 group compared to A30 group (323 ± 161 *vs*. 224 ± 64 mg/dl). Other parameters including HCO_3_, PO_2_, and PCO_2_, also showed a significant difference in A30 and A60 when compared to PC group (Table 1, *p* < 0.05), and in A60 (Table 1, A60 *vs*. A30, *p <* 0.05).

### Effect of cREBOA duration on circulating CBC and coagulation

**Fig 3:**
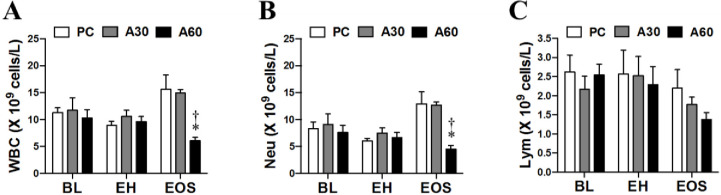
White blood cell counts in swine undergoing cREBOA. Data are presented as mean ± SEM and were statistically analyzed using two-tailed Mann-Whitney test. * *p*<0.05, PC *vs*. A60 group; † *p*<0.05, A60 *vs*. A30 group. WBC, white blood cells; Neu, neutrophils; Lym, lymphocytes.

**Fig 4:**
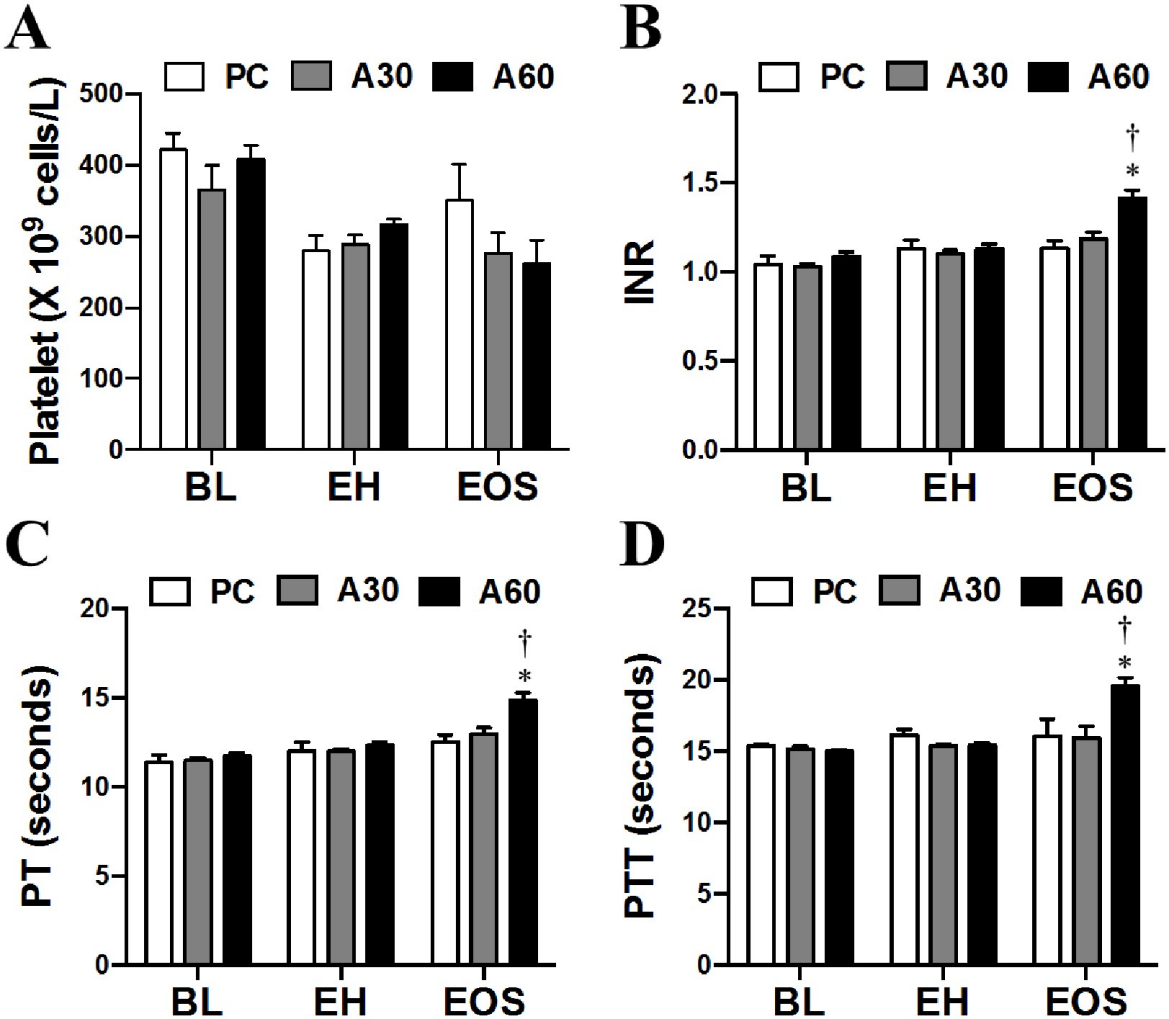
Blood clotting measures in swine undergoing cREBOA. Data are presented as mean ± SEM and were statistically analyzed using two-tailed Mann-Whitney test. * *p*<0.05, PC *vs*. A60 group; † *p*<0.05, A60 *vs*. A30 group. INR, international normalized ratio; PT, prothrombin time; PTT, partial thromboplastin time.

As shown in Figs 3 and 4, there was no significant difference in white blood cells (WBCs, neutrophils, lymphocytes, platelets, INR, PT, and PTT in all groups at BL and EH (*p >* 0.05). Circulating number of WBCs (Fig 3A) and neutrophils (Fig 3B) in A60 at EOS was significantly lower by 3- and 2-fold than PC and A30 respectively (*p* < 0.05). Lymphocyte counts were less in A60 at EOS compared to PC, but no significant differences were noted (Fig 3C) *p* > 0.05). The platelet count decreased in both A30 and A60 groups compared with PC, but did not reach statistical difference (Fig 4A *p >* 0.05)). Significantly prolonged INR, PT and PTT in A60 at EOS were observed when compared to PC and A30 (Fig 4B–4D, *p* < 0.05).

### Effect of cREBOA duration on tissue inflammatory cytokines and oxidative stress

**Fig 5:**
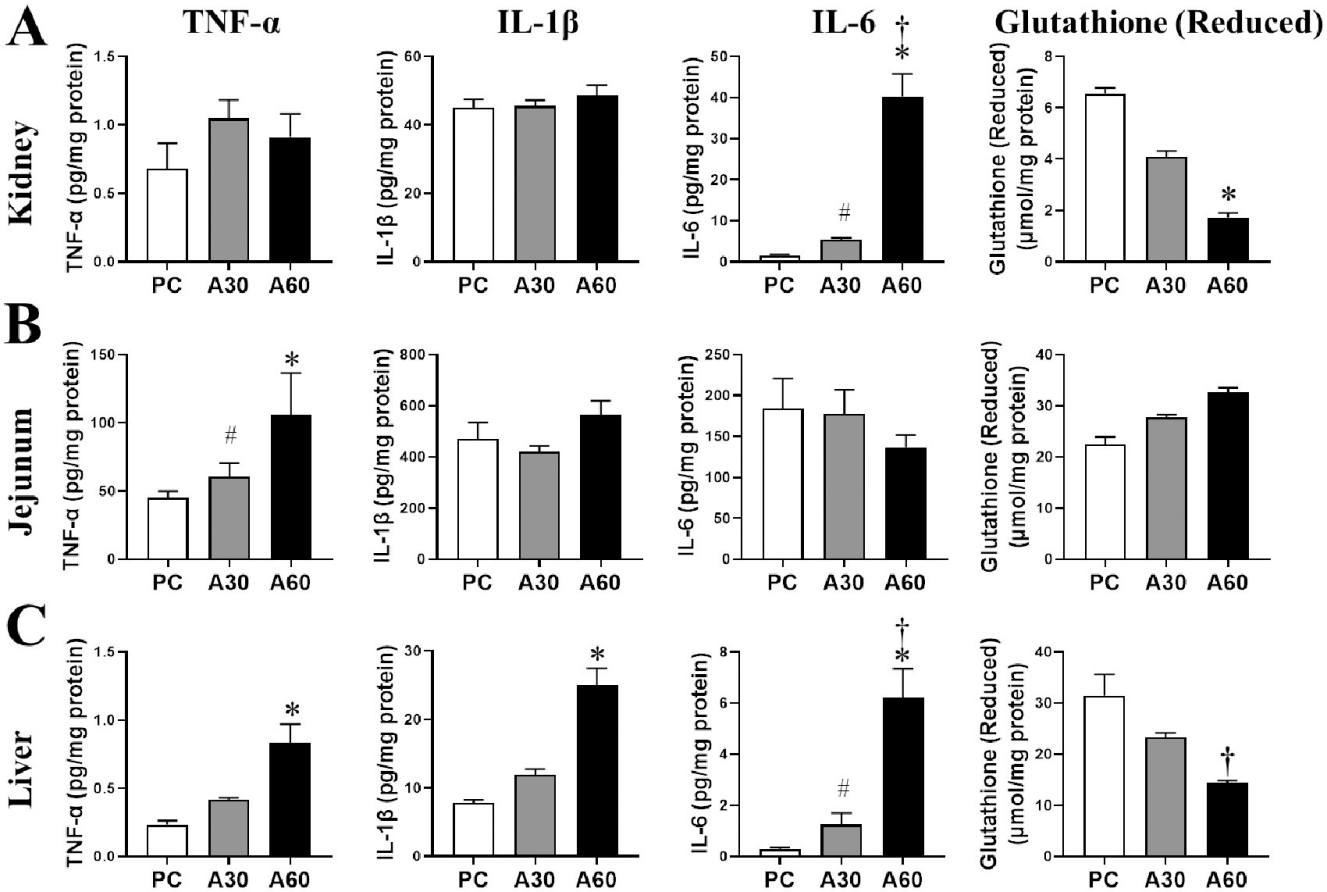
Local tissue inflammatory response and oxidative stress in swine undergoing cREBOA. Data are presented as mean ± SEM and were statistically analyzed using two-tailed Mann-Whitney test. ^#^ *p*<0.05, A30 *vs*. PC; * *p*<0.05, A60 *vs*. PC; † *p*<0.05, A60 *vs*. A30.

Both A30 and A60 showed significant increase in renal IL-6, intestinal TNF-α, and hepatic IL-6 (*vs*. PC, *p <* 0.05, Fig 5A–5C), while higher levels of IL-6 in both kidney and liver tissues were observed in A60 group when compared to the A30 group (*p <* 0.05, Fig 5A and 5C). There were significant elevated tissue levels of TNF-α and IL-1β in liver compared with the PC (*p <* 0.05, Fig 5C). No significant difference was detected in renal TNF-α and IL-1β, and intestinal hepatic IL-1β and IL-6 within the 3 groups (*p* >0.05, Fig 5A–5C). No significant change of renal, hepatic and intestinal IL-10 was observed among the 3 groups.

Next we analyzed reduced glutathione in different tissue isolated from the animals of the cREBOA and PC groups. Our analysis revealed significant decrease in renal (Fig 5A) and hepatic (Fig 4C) reduced glutathione in A60 when compared with PC or A30 respectively (p < 0.05), but there were no statistical differences of intestinal reduced glutathione in both A30 and A60 when compared with PC (*p* > 0.05, Fig 5B).

### Effect of cREBOA duration on circulating end organ damage markers

**Fig 6:**
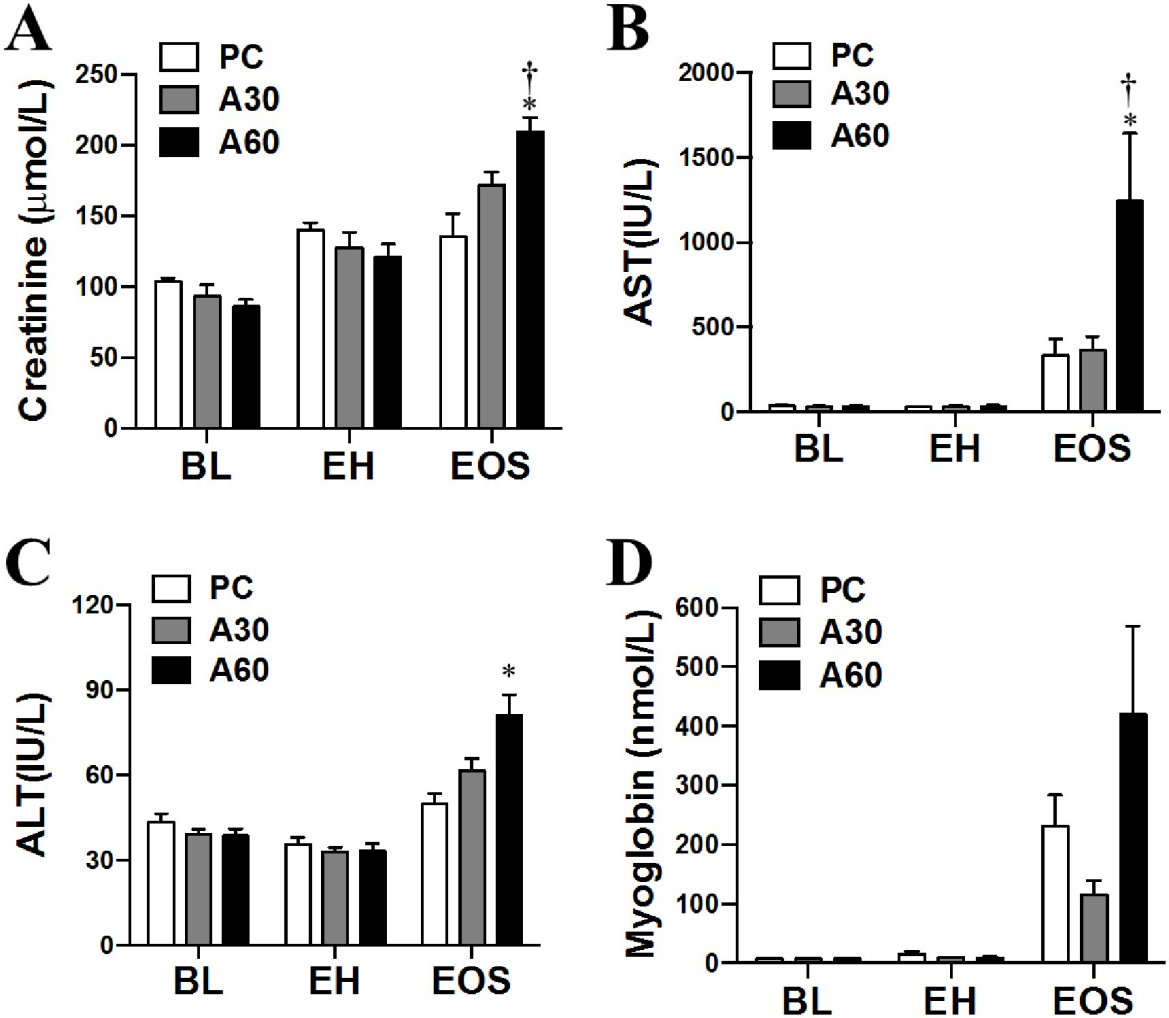
Changes of end-organ damage in plasma from swine undergoing cREBOA. Data are presented as mean ± SEM and were statistically analyzed using two-tailed Mann-Whitney test. * *p*<0.05, PC *vs*. A60 group; † *p*<0.05, A60 *vs*. A30 group. ALT, alanine aminotransferase; AST, aspartate aminotransferase.

As shown in Fig 6, the organ damage profile across the 3 groups was very similar at BL and EH (*p >* 0.05). Significant increases in plasma creatinine concentrations were detected at EOS in A60 compared to both A30 and PC (*p* < 0.05, Fig 6A). Both AST and ALT concentrations markedly higher in A60 compared to PC (*p* < 0.05, Fig 6B–6C), whereas only significant difference in AST was found at EOS in A60 compared to A30 (*p <* 0.05, Fig 6B). In the case of myoglobin, although markedly higher in A60 compared to A30, no significant difference was observed at EOS (*p >* 0.05, Fig 6D).

### Effect of cREBOA duration on histopathological alterations

**Fig 7:**
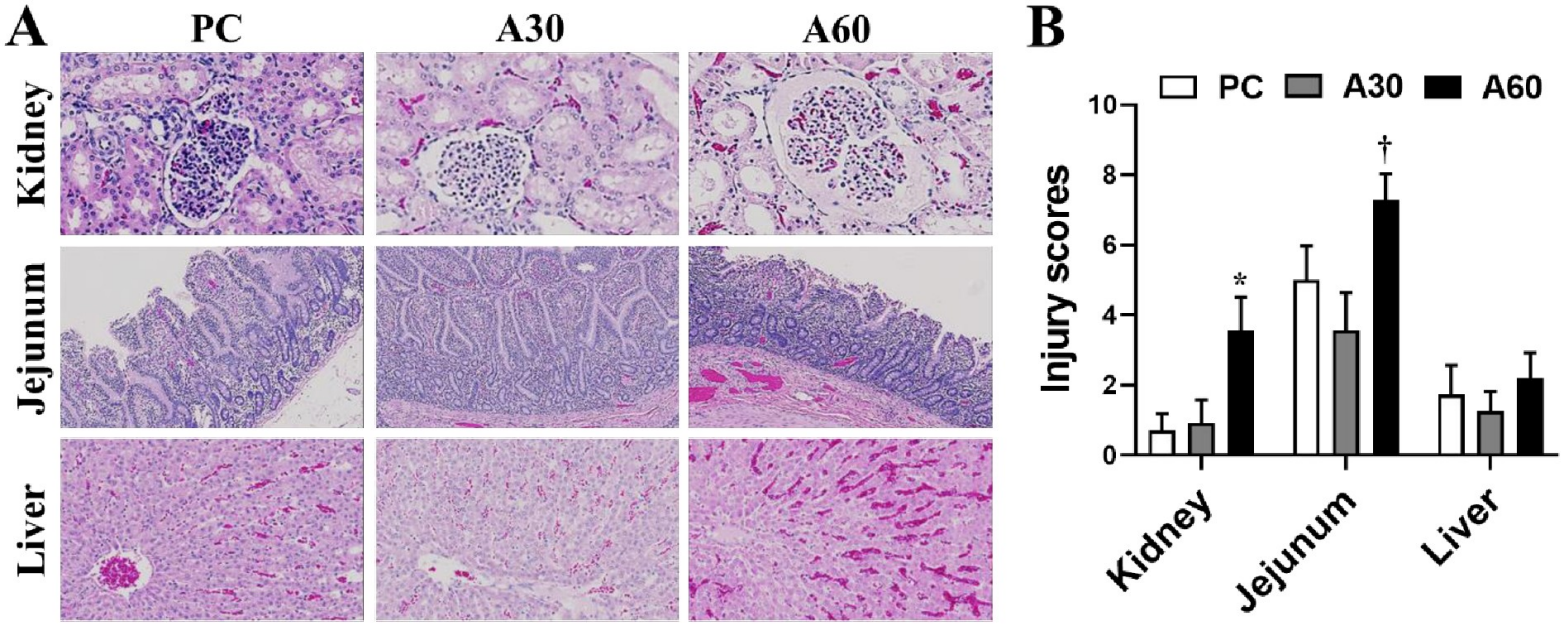
Histological alterations of end-organs in swine undergoing cREBOA. A, representative histological photos of kidney (top panel), jejunum (middle panel) and liver (low panel). B, semiquantitative evaluation of the pathological features. Data are presented as mean ± SEM and were statistically analyzed using two-tailed Mann-Whitney test. * *p*<0.05, PC *vs*. A60 group; † *p*<0.05, A60 *vs*. A30 group.

Histopathology evaluation of the distal organ injury was performed at the EOS (Fig 7). Renal injury characterized by dilated Bowman’s space filled with hyaline materials, intensive vacuolization and necrotic/apoptotic cells in renal tubules, interstitial edema and inflammatory cell infiltration, and thrombosis/congestion, and semi-quantitative histopathological evaluation showed significantly higher injury score in A60 than PC (p < 0.05). Intestinal tissue of the A60 group presented with significant damage featured by villus hemorrhage and denudation, lamina propria inflammatory cell infiltration, and congestion and exudation along with an increased injury score when compared with A30 (*p* < 0.05). There was no significant difference in histological injury score in the liver tissue among the 3 groups (*p* > 0.05), but greater congestion, dilation, and leukocyte sequestration within hepatic sinusoids were observed in the A60 group compared to the A30 and PC. Semi-quantitative histopathology evaluation of brain showed a trend of improved injury scores in the cerebral cortex (A30: 0.68 ± 0.44, A60: 0.82 ± 0.32) and hypothalamus (A30: 1 ± 0.37, A60: 1.23 ± 0.4) after the cREBOA when compared with the changes in the cerebral cortex (1.32 ± 0.55) and hypothalamus (1.59 ± 0.53) of the animals in the PC group (*p* > 0.05).

## Discussion

Previously, several studies addressed cREBOA-associated ischemia reperfusion injury (IRI) sequelae including systemic inflammation, metabolic acidosis, and organ dysfunction after a prolonged cREBOA use in hemorrhagic shock models in anesthetized pigs [24–27], baboons [28], and sheep [29]. However, there were unanswered questions in terms of cREBOA balloon inflation time and effects on distal organ inflammation, function and injury. In this current study, we sought to evaluate these questions regarding aortic balloon inflation times in a conscious and sedated porcine severe hemorrhagic shock model. The principal findings were as follows: compared with 30min-cREBOA group, 60min-cREBOA demonstrated (1) significantly greater systemic and distal organ inflammatory response, and reduction in reduced glutathione in kidney, jejunum and liver; (2) significant multiple-end organ dysfunction and injury; and (3) disturbances of hemodynamics and metabolism as well as coagulation.

Rasmussen and his colleagues [10, 22, 30–32] demonstrated that cREBOA has emerged as an effective life-saving maneuver for the management of non-compressible torso hemorrhage in trauma patients. cREBOA is inherently associated with the burden of lower body IRI and possible organ dysfunction. Therefore, the use of cREBOA is limited by the burden of IRI to distal organs. Indeed, as mentioned others have addressed cREBOA-associated IRI sequelae including systemic inflammation, metabolic acidosis, and organ dysfunction after in several animal hemorrhagic shock models [21–26]. Consistent with these findings, the current data revealed that prolonged cREBOA (60min balloon inflation) after hemorrhage led to distal tissue hypoperfusion reflected by hypotension from R90 to the EOS and the higher lactate and lower BE from 30min to the EOS after the occlusion indicating a longer ischemia time. The hypoperfusion after the prolonged cREBOA appears to contribute to distal multiple-organ dysfunction (kidney, liver, and muscle) and damage (kidney and jejunum), hyperkalemia, and coagulation alterations. The hypotension in A60 group may be caused by reduced cardiac output as a result of hyperkalemia and/or preload reduction due to IRI-induced hyperemia reflected by increased tissue congestion in the distal organs (kidney, jejunum and liver).

Although previous studies demonstrated that prolonged cREBOA time resulted in a systemic inflammatory response in swine [24] and non-human primate [28], tissue inflammatory response to prolonged cREBOA after hemorrhage in the distal organs has not been investigated. The enhanced release of the inflammatory cytokines TNF-α, IL-1β, and IL-6 are the biomarkers of IRI [33]. The most noteworthy finding in the current study was tissue inflammatory response in tissues as evidenced by greater levels of IL-6 in kidney, TNF-α in jejunum, and TNF-α, IL-1β and IL-6 in liver in the 60min-cREBOA group when compared to the PC or the 30min-cREBOA group. Our findings of highly elevated pro-inflammatory cytokines in the organs below the aortic occlusion indicate that these tissues underwent IRI. Recent studies illustrated glutathione derangement and stress hyperglycemia in the settings of tissue injury/hemorrhage and critical illness [34, 35]. Reduced glutathione is a powerful intracellular antioxidant that can modulate NF-κB and AP-1 activation influencing transcription of cytokines and adhesion molecules [36]. In this study, decreased levels of reduced glutathione in kidney and liver after 60min-cREBOA may at least partially contribute to the tissue cytokine production and organ damage observed.

In contrast to a previous report [28], the 60min cREBOA group in this current study had acute leukopenia, neutropenia, and platelet decline at the EOS. Acute leukopenia is a recognized condition in critically ill septic and cancer patients [37, 38]. The exact mechanism of leukopenia and platelet reduction after cREBOA and hemorrhage is unknown, but there are interesting speculations. According to previous studies [39, 40], we generally assume that the acute leukopenia/neutropenia and platelet reduction after 60min-cREBOA in this study are probably due to IRI-induced (1) leukocyte/platelet activation and sequestration, and/or (2) overwhelming bacterial translocation from the damaged intestine. Indeed, upregulation of neutrophil integrin CD11b after supraceliac aortic clamping in patients was shown as the primary adhesive glycoprotein responsible for neutrophil tissue entrapment and neutrophil-mediated reperfusion injury [41]. Moreover, histological evidence of liver, jejunum, and kidney injury reflected by vascular congestion and inflammatory cell infiltration indicate that leukocyte/platelet activation and sequestration are the most likely mechanism for leukopenia and platelet reduction in this study. The leukocyte sequestration in the injured tissues may contribute to the increase in levels of TNF-α, IL-1β, and IL-6 in liver, kidney and small intestine. Our finding is consistent with the previous report that neutrophil infiltration into IRI tissues is involved in upregulation of hepatic inflammatory proteins, the complement system, chemokine, oxidative stress, and increased capillary permeability [42, 43]. Uncontrolled inflammation is often accompanied by microvascular thrombosis. Several inflammatory cytokines such as TNF-α may activate tissue factor and coagulation [44] as we have shown in our study with the 60min cREBOA treatment. Unlike previous reports [24, 28], lack of clear changes in the blood cytokines (IL-1β, IL-6, IL-8, IL-10, and TNF-α) in this study may simply reflect different experimental conditions as species, preoperative status, differences in surgical extensity, the methods of aortic occlusion and subject management, and a short follow-up.

Below the aortic occlusion, IRI primarily affects endothelium-dependent vasodilation other than vasoconstriction, a state known as “reactive hyperemia” contributing to reduction in afterload that may result in distributive shock [45, 46]. In accordance with it, the 60min cREBOA time in the current study led to increased tissue congestion and edema in the distal organs (kidney, jejunum and liver) compared with the 30min-cREBOA group. These alterations may explain, at least in part, why the 60min-cREBOA group had significantly higher levels of Hb and Hct, and lower blood pressures post-cREBOA deflation.

Numerous studies in critically ill patients have demonstrated that stress hyperglycemia is associated with poor clinical outcomes. Although the acute treatment of mild-moderate hyperglycemia with intensive glucose control in acutely ill patients lacks biologic plausibility [47], persistent severe stress hyperglycemia may lead to its effects on osmolarity-induced fluid shift, diuresis and volume depletion [35]. In this study, severe hyperglycemia (60min-cREBOA *vs*. 30min-cREBOA: 323 mg/dl *vs*. 224 mg/dl) may be harmful, which may partially contribute to the organ dysfunction and damage. Therefore, persistent severe stress hyperglycemia may benefit from moderate glycemic control in our future cREBOA studies.

It is becoming evident that multiple-organ crosstalk after IRI plays an important role in multiple-organ dysfunction [48]. Recent insights in basic and clinical research have been gained into organ-organ crosstalk, which contributes to metabolic homeostasis, inflammatory response, endothelial injury, oxidative stress and reactive oxygen species production, apoptosis, and coagulopathy during sepsis and acute kidney injury (AKI) [49–51]. Although liver tissue is relatively resistant to IRI itself, this condition triggers the generation of many factors including cytokines and chemokines produced by Kupffer cells (KCs), anaerobic metabolism, mitochondrial damage, oxidative stress, and intracellular Ca^2+^ overload, which cause acute liver failure (ALF) as well as commonly lead to extrahepatic multiple-organ dysfunction (MOD) [52, 53]. Indeed, liver tissue generation of cytokines (IL-1β and TNF-α) produced by KCs precedes the late, cellular phase of reperfusion injury [54, 55]. Generally, AKI occurs often in patients with acute liver injury [56]. Hypotension leads to up-regulation of renin-angiotensin system that can severally reduce glomerular filtration rate, urinary sodium excretion, and free water excretion. KCs are activated after liver IRI and predominantly contribute to increased circulating levels of pro-inflammatory cytokines, including IL-6, TNF-α, and high-mobility group box 1 protein and their release from the liver tissue can initiate inflammation in the kidney tissue [54]. In the current study, the plasma myoglobin concentration was higher in A60 than in the A30 group, indicating A60 as a stronger rhabdomyolysis case. Acute renal injury appears as a complication of myoglobinuria in the setting of any trauma [57, 58]. Rhabdomyolysis that is associated with the A60 group could contribute to the pathogenesis of AKI observed in our study.

Intestinal IRI disrupts mucosal integrity and increases intestinal permeability resulting in dysbiosis, and translocation of bowel bacteria and their endotoxins into the circulation that lead to systemic inflammation, and immune, metabolic and coagulation alteration [59]. This altered homeostasis may mediate and potentiate the development of MOD including AKI and ALF [49, 60, 61]. In turn, the development of AKI and ALF decreases the clearance of inflammatory mediators, metabolic products, and translocated bacteria and their endotoxins, leading to a vicious cycle and further gut injury. It may also have a role in the pathogeneses of coagulopathy, along with hepatic ischemia. Increased plasma concentrations of cytokines (e.g. TNF-α, IL-6, IL-8, and IL-10) are often observed in patients with aortic cross-clamping/ischemia longer than 40 minutes [62, 63]. In our porcine study, cREBOA treatment for 60 minutes did induce significant damage to the intestinal tissue, but displayed no significant changes of IL-1β, IL-6, and oxidative stress unlike kidney and liver tissue. Plausible explanations are that these contradictory data may be due to a different kinetics of the pro-inflammatory cytokines/glutathione production in jejunum in response to IRI, and/or loss of villi where are heavily populated with activated leukocytes after IRI. Similarly, liver enzymes (AST and ALT) increased to abnormal concentrations with no significant differences in liver tissue damage even though oxidative stress was severe. These results demonstrate that reduced glutathione or AST and ALT may be not reliable indicators of intestinal and liver damage, respectively. High-mobility group box 1 protein, matrix metalloproteinases, neutrophil gelatinase-associated lipocalin, diamine oxidase, fatty acid-binding proteins, and citrulline could be used as new biomarkers of IRI of intestine and liver [52, 64].

Therefore, in order to mitigate the prolonged cREBOA-induced IRI, It will be worthwhile to consider different treatment approaches as follows: (1) immunomodulation, (2) metabolic therapeutic interventions, (3) intermittent infusion of oxygenated fluid/blood, and (4) self-regulated partial REBOA.

## Limitations

Our study does not include spinal cord effects that may yield new challenges. Because interaction among damage-associated molecular patterns, complement cascade, and inflammasome plays a pivotal role in tissue IRI [65], the study of these cascades deserves attention in highlighting their importance. Since different organs may have different time windows of the inflammatory response to IRI, future study will be needed to determine the kinetics of the tissue inflammation during cREBOA. Finally, the observation period of this study is too short to determine a long-term impact on survival, inflammatory response and distal organ damage, longer follow-up should be carried out in future.

## Conclusions

Although life-saving in 100% of animals, cREBOA treatment of severe hemorrhage should be shorter than 60 minutes due to its impact on pathophysiological sequelae, specifically on distal organ inflammation and damage. In all, cREBOA application deserves further research that would include mitigation of inflammation, coagulopathy, distal organ IRI, and longer follow-up.

## Supporting information

S1 Table Vital signs of individual animals. (DOCX)

## References

[1] Kragh JF Jr., Walters TJ, Baer DG, Fox CJ, Wade CE, Salinas J, et al Survival with emergency tourniquet use to stop bleeding in major limb trauma. Ann Surg. 2009;249(1):1–7. Epub 2008/12/25. 10.1097/SLA.0b013e31818842ba . 19106667

[2] Kheirabadi BS, Scherer MR, Estep JS, Dubick MA, Holcomb JB. Determination of efficacy of new hemostatic dressings in a model of extremity arterial hemorrhage in swine. J Trauma. 2009;67(3):450–9; discussion 9–60. Epub 2009/09/11. 10.1097/TA.0b013e3181ac0c99 . 19741385

[3] Butler FK Jr., Holcomb JB, Shackelford S, Barbabella S, Bailey JA, Baker JB, et al Advanced Resuscitative Care in Tactical Combat Casualty Care: TCCC Guidelines Change 18–01:14 October 2018. J Spec Oper Med. 18(4):37–55. . 30566723 10.55460/YJB8-ZC0Y

[4] Stannard A, Morrison JJ, Scott DJ, Ivatury RA, Ross JD, Rasmussen TE. The epidemiology of noncompressible torso hemorrhage in the wars in Iraq and Afghanistan. J Trauma Acute Care Surg. 2013;74(3):830–4. Epub 2013/02/22. 10.1097/TA.0b013e31827a3704 . 23425743

[5] Babbs CF. Hemodynamic mechanisms in CPR: a theoretical rationale for resuscitative thoracotomy in non-traumatic cardiac arrest. Resuscitation. 1987;15(1):37–50. Epub 1987/03/01. 10.1016/0300-9572(87)90096-7 . 3035669

[6] Vij D, Simoni E, Smith RF, Obeid FN, Horst HM, Tomlanovich MC, et al Resuscitative thoracotomy for patients with traumatic injury. Surgery. 1983;94(4):554–61. Epub 1983/10/01. . 6623356

[7] White JM, Cannon JW, Stannard A, Markov NP, Spencer JR, Rasmussen TE. Endovascular balloon occlusion of the aorta is superior to resuscitative thoracotomy with aortic clamping in a porcine model of hemorrhagic shock. Surgery. 2011;150(3):400–9. Epub 2011/09/01. 10.1016/j.surg.2011.06.010 . 21878225

[8] Avaro JP, Mardelle V, Roch A, Gil C, de Biasi C, Oliver M, et al Forty-minute endovascular aortic occlusion increases survival in an experimental model of uncontrolled hemorrhagic shock caused by abdominal trauma. J Trauma. 2011;71(3):720–5; discussion 5–6. Epub 2011/09/13. 10.1097/TA.0b013e318221a94a . 21909002

[9] Markov NP, Percival TJ, Morrison JJ, Ross JD, Scott DJ, Spencer JR, et al Physiologic tolerance of descending thoracic aortic balloon occlusion in a swine model of hemorrhagic shock. Surgery. 2013;153(6):848–56. Epub 2013/03/05. 10.1016/j.surg.2012.12.001 . 23453327

[10] Stannard A, Eliason JL, Rasmussen TE. Resuscitative endovascular balloon occlusion of the aorta (REBOA) as an adjunct for hemorrhagic shock. J Trauma. 2011;71(6):1869–72. Epub 2011/12/21. 10.1097/TA.0b013e31823fe90c . 22182896

[11] Manzano Nunez R, Ordonez Delgado CA. Analysis of REBOA in systematic reviews: it is early to provide evidence-based, strong recommendations. Eur J Trauma Emerg Surg. 2017;43(2):281–2. Epub 2017/03/11. 10.1007/s00068-017-0763-0 . 28280875

[12] Borger van der Burg BLS, van Dongen T, Morrison JJ, Hedeman Joosten PPA, DuBose JJ, Horer TM, et al A systematic review and meta-analysis of the use of resuscitative endovascular balloon occlusion of the aorta in the management of major exsanguination. Eur J Trauma Emerg Surg. 2018;44(4):535–50. Epub 2018/05/23. 10.1007/s00068-018-0959-y . 29785654 PMC6096615

[13] Maj Jason Pasley U, MC, Lt Col Jeremy Cannon U, MC CDR Jacob Glaser, MC, USN, CDR Travis Polk M, USN, MAJ Jonathan Morrison RMJB, USAF, MC, Lt Col Benjamin Mitchell U, MC, Maj Justin Manley U, MC LTC Tyson Becker, MC, USA, et al. Resuscitative Endovascular Balloon Occlusion of the Aorta (REBOA) for Hemorrhagic Shock (CPG ID: 38). JOINT TRAUMA SYSTEM CLINICAL PRACTICE GUIDELINE (JTS CPG). 2014.

[14] Park TS, Batchinsky AI, Belenkiy SM, Jordan BS, Baker WL, Necsoiu CN, et al Resuscitative endovascular balloon occlusion of the aorta (REBOA): Comparison with immediate transfusion following massive hemorrhage in swine. J Trauma Acute Care Surg. 2015;79(6):930–6. Epub 2015/12/19. 10.1097/TA.0000000000000877 . 26680136

[15] Dunn EL, Moore EE, Moore JB. Hemodynamic effects of aortic occlusion during hemorrhagic shock. Ann Emerg Med. 1982;11(5):238–41. Epub 1982/05/01. 10.1016/s0196-0644(82)80090-5 . 7041702

[16] Sankaran S, Lucas C, Walt AJ. Thoracic aortic clamping for prophylaxis against sudden cardiac arrest during laparotomy for acute massive hemoperitoneum. J Trauma. 1975;15(4):290–6. Epub 1975/04/01. 10.1097/00005373-197504000-00005 . 1127755

[17] Mitteldorf C, Poggetti RS, Zanoto A, Branco PD, Birolini D, Castro de Tolosa EM, et al Is aortic occlusion advisable in the management of massive hemorrhage? Experimental study in dogs. Shock. 1998;10(2):141–5. Epub 1998/08/29. 10.1097/00024382-199808000-00010 . 9721982

[18] Gelman S. The pathophysiology of aortic cross-clamping and unclamping. Anesthesiology. 1995;82(4):1026–60. Epub 1995/04/01. 10.1097/00000542-199504000-00027 . 7717537

[19] Cruz RJ Jr., Poli de Figueiredo LF, Bras JL, Rocha e Silva M. Effects of intra-aortic balloon occlusion on intestinal perfusion, oxygen metabolism and gastric mucosal PCO2 during experimental hemorrhagic shock. Eur Surg Res. 2004;36(3):172–8. Epub 2004/06/05. 10.1159/000077260 . 15178907

[20] Kauvar DS, Dubick MA, Martin MJ. Large Animal Models of Proximal Aortic Balloon Occlusion in Traumatic Hemorrhage: Review and Identification of Knowledge Gaps Relevant to Expanded Use. J Surg Res. 2019;236:247–58. 10.1016/j.jss.2018.11.038 . 30694763

[21] Burns JW, Baer LA, Hagerman EJ, Jordan BS, Nelson JJ Jr., Batchinsky AI, et al Development and resuscitation of a sedated, mature male miniature swine severe hemorrhage model. J Trauma. 2011;71(1):148–56. Epub 2010/11/09. 10.1097/TA.0b013e3181eaaf6b . 21057337

[22] Scott DJ, Eliason JL, Villamaria C, Morrison JJ, Houston Rt, Spencer JR, et al A novel fluoroscopy-free, resuscitative endovascular aortic balloon occlusion system in a model of hemorrhagic shock. J Trauma Acute Care Surg. 2013;75(1):122–8. Epub 2013/08/14. 10.1097/ta.0b013e3182946746 . 23940855

[23] Scaravilli V, Kreyer S, Belenkiy S, Linden K, Zanella A, Li Y, et al Extracorporeal Carbon Dioxide Removal Enhanced by Lactic Acid Infusion in Spontaneously Breathing Conscious Sheep. Anesthesiology. 2016;124(3):674–82. Epub 2016/01/13. 10.1097/ALN.0000000000000995 . 26756517

[24] Morrison JJ, Ross JD, Markov NP, Scott DJ, Spencer JR, Rasmussen TE. The inflammatory sequelae of aortic balloon occlusion in hemorrhagic shock. J Surg Res. 2014;191(2):423–31. Epub 2014/05/20. 10.1016/j.jss.2014.04.012 . 24836421

[25] Sadeghi M, Horer TM, Forsman D, Dogan EM, Jansson K, Kindler C, et al Blood pressure targeting by partial REBOA is possible in severe hemorrhagic shock in pigs and produces less circulatory, metabolic and inflammatory sequelae than total REBOA. Injury. 2018;49(12):2132–41. Epub 2018/10/12. 10.1016/j.injury.2018.09.052 . 30301556

[26] Hoehn MR, Teeter WA, Morrison JJ, Gamble WB, Hu P, Stein DM, et al Aortic branch vessel flow during resuscitative endovascular balloon occlusion of the aorta. J Trauma Acute Care Surg. 2019;86(1):79–85. Epub 2018/09/27. 10.1097/TA.0000000000002075 . 30252777

[27] Tibbits EM, Hoareau GL, Simon MA, Davidson AJ, DeSoucy ES, Faulconer ER, et al Location is everything: The hemodynamic effects of REBOA in Zone 1 versus Zone 3 of the aorta. J Trauma Acute Care Surg. 2018;85(1):101–7. Epub 2018/07/03. 10.1097/TA.0000000000001858 . 29965941

[28] Eliason JL, Myers DD, Ghosh A, Morrison JJ, Mathues AR, Durham L, et al Resuscitative Endovascular Balloon Occlusion of the Aorta (REBOA): Zone I Balloon Occlusion Time Affects Spinal Cord Injury in the Nonhuman Primate Model. Ann Surg. 2019 Epub 2019/06/13. 10.1097/SLA.0000000000003408 . 31188208

[29] Reva VA, Matsumura Y, Horer T, Sveklov DA, Denisov AV, Telickiy SY, et al Resuscitative endovascular balloon occlusion of the aorta: what is the optimum occlusion time in an ovine model of hemorrhagic shock? Eur J Trauma Emerg Surg. 2018;44(4):511–8. Epub 2016/10/16. 10.1007/s00068-016-0732-z . 27738726

[30] Davidson AJ, Russo RM, Ferencz SE, Cannon JW, Rasmussen TE, Neff LP, et al Incremental balloon deflation following complete resuscitative endovascular balloon occlusion of the aorta results in steep inflection of flow and rapid reperfusion in a large animal model of hemorrhagic shock. J Trauma Acute Care Surg. 2017;83(1):139–43. Epub 2017/06/21. 10.1097/TA.0000000000001502 . 28632583 PMC5484091

[31] Morrison JJ, Ross JD, Houston Rt, Watson JD, Sokol KK, Rasmussen TE. Use of resuscitative endovascular balloon occlusion of the aorta in a highly lethal model of noncompressible torso hemorrhage. Shock. 2014;41(2):130–7. Epub 2014/01/17. 10.1097/SHK.0000000000000085 . 24430492

[32] Morrison JJ, Percival TJ, Markov NP, Villamaria C, Scott DJ, Saches KA, et al Aortic balloon occlusion is effective in controlling pelvic hemorrhage. J Surg Res. 2012;177(2):341–7. Epub 2012/05/18. 10.1016/j.jss.2012.04.035 . 22591921

[33] Huber-Lang M, Lambris JD, Ward PA. Innate immune responses to trauma. Nat Immunol. 2018;19(4):327–41. Epub 2018/03/07. 10.1038/s41590-018-0064-8 . 29507356 PMC6027646

[34] Slaughter AL, Nunns GR, D’Alessandro A, Banerjee A, Hansen KC, Moore EE, et al The Metabolopathy of Tissue Injury, Hemorrhagic Shock, and Resuscitation in a Rat Model. Shock. 2018;49(5):580–90. Epub 2017/07/21. 10.1097/SHK.0000000000000948 . 28727610 PMC5775055

[35] Marik PE, Bellomo R. Stress hyperglycemia: an essential survival response! Crit Care. 2013;17(2):305 Epub 2013/03/09. 10.1186/cc12514 . 23470218 PMC3672537

[36] Biswas SK, Rahman I. Environmental toxicity, redox signaling and lung inflammation: the role of glutathione. Mol Aspects Med. 2009;30(1–2):60–76. Epub 2008/09/02. 10.1016/j.mam.2008.07.001 . 18760298 PMC2699458

[37] Georges Q, Azoulay E, Mokart D, Soares M, Jeon K, Oeyen S, et al Influence of neutropenia on mortality of critically ill cancer patients: results of a meta-analysis on individual data. Crit Care. 2018;22(1):326 Epub 2018/12/06. 10.1186/s13054-018-2076-z . 30514339 PMC6280476

[38] Patel A, Gruber P. Severe infections in neutropenic patients. Curr Opin Crit Care. 2015;21(6):586–92. Epub 2015/11/06. 10.1097/MCC.0000000000000256 . 26539933

[39] Ing VW. The etiology and management of leukopenia. Can Fam Physician. 1984;30:1835–9. Epub 1984/09/01. . 21279100 PMC2154209

[40] Ardizzone G, Stratta C, Valzan S, Crucitti M, Gallo M, Cerutti E. Acute blood leukocyte reduction after liver reperfusion: a marker of ischemic injury. Transplant Proc. 2006;38(4):1076–7. Epub 2006/06/08. 10.1016/j.transproceed.2006.02.011 . 16757269

[41] Hill GE, Mihalakakos PJ, Spurzem JR, Baxter TB. Supraceliac, but not infrarenal, aortic cross-clamping upregulates neutrophil integrin CD11b. J Cardiothorac Vasc Anesth. 1995;9(5):515–8. Epub 1995/10/01. 10.1016/s1053-0770(05)80133-8 . 8547551

[42] Zhang P, Xie M, Zagorski J, Spitzer JA. Attenuation of hepatic neutrophil sequestration by anti-CINC antibody in endotoxic rats. Shock. 1995;4(4):262–8. 10.1097/00024382-199510000-00006 . 8564554

[43] Garcia-Criado FJ, Palma-Vargas JM, Valdunciel-Garcia JJ, Toledo AH, Misawa K, Gomez-Alonso A, et al Tacrolimus (FK506) down-regulates free radical tissue levels, serum cytokines, and neutrophil infiltration after severe liver ischemia. Transplantation. 1997;64(4):594–8. 10.1097/00007890-199708270-00008 . 9293871

[44] Barie PS, Hydo LJ, Pieracci FM, Shou J, Eachempati SR. Multiple organ dysfunction syndrome in critical surgical illness. Surg Infect (Larchmt). 2009;10(5):369–77. Epub 2009/12/01. 10.1089/sur.2009.9935 . 19943773

[45] Levin A. The cardiovascular effects of aortic clamping and unclamping. Southern African Journal of Anaesthesia and Analgesia. 2010;16(2):62–71.

[46] Gourdin MJ, Bree B, De Kock M. The impact of ischaemia-reperfusion on the blood vessel. Eur J Anaesthesiol. 2009;26(7):537–47. Epub 2009/05/05. 10.1097/EJA.0b013e328324b7c2 . 19412112

[47] Investigators N-SS, Finfer S, Chittock DR, Su SY, Blair D, Foster D, et al Intensive versus conventional glucose control in critically ill patients. N Engl J Med. 2009;360(13):1283–97. Epub 2009/03/26. 10.1056/NEJMoa0810625 . 19318384

[48] Armutcu F. Organ crosstalk: the potent roles of inflammation and fibrotic changes in the course of organ interactions. Inflamm Res. 2019;68(10):825–39. Epub 2019/07/22. 10.1007/s00011-019-01271-7 . 31327029

[49] Lee SA, Cozzi M, Bush EL, Rabb H. Distant Organ Dysfunction in Acute Kidney Injury: A Review. Am J Kidney Dis. 2018;72(6):846–56. Epub 2018/06/06. 10.1053/j.ajkd.2018.03.028 . 29866457 PMC6252108

[50] Lelubre C, Vincent JL. Mechanisms and treatment of organ failure in sepsis. Nat Rev Nephrol. 2018;14(7):417–27. Epub 2018/04/25. 10.1038/s41581-018-0005-7 . 29691495

[51] Li X, Hassoun HT, Santora R, Rabb H. Organ crosstalk: the role of the kidney. Curr Opin Crit Care. 2009;15(6):481–7. Epub 2009/10/24. 10.1097/MCC.0b013e328332f69e . 19851101

[52] Cannistra M, Ruggiero M, Zullo A, Gallelli G, Serafini S, Maria M, et al Hepatic ischemia reperfusion injury: A systematic review of literature and the role of current drugs and biomarkers. Int J Surg. 2016;33 Suppl 1:S57–70. Epub 2016/06/04. 10.1016/j.ijsu.2016.05.050 . 27255130

[53] Wadei HM, Lee DD, Croome KP, Mai ML, Golan E, Brotman R, et al Early Allograft Dysfunction After Liver Transplantation Is Associated With Short- and Long-Term Kidney Function Impairment. Am J Transplant. 2016;16(3):850–9. Epub 2015/12/15. 10.1111/ajt.13527 . 26663518

[54] Nastos C, Kalimeris K, Papoutsidakis N, Tasoulis MK, Lykoudis PM, Theodoraki K, et al Global consequences of liver ischemia/reperfusion injury. Oxid Med Cell Longev. 2014;2014:906965 Epub 2014/05/07. 10.1155/2014/906965 . 24799983 PMC3995148

[55] Colletti LM, Green M. Lung and liver injury following hepatic ischemia/reperfusion in the rat is increased by exogenous lipopolysaccharide which also increases hepatic TNF production in vivo and in vitro. Shock. 2001;16(4):312–9. Epub 2001/10/03. 10.1097/00024382-200116040-00014 . 11580116

[56] Davenport A, Sheikh MF, Lamb E, Agarwal B, Jalan R. Acute kidney injury in acute-on-chronic liver failure: where does hepatorenal syndrome fit? Kidney Int. 2017;92(5):1058–70. 10.1016/j.kint.2017.04.048 . 28844314

[57] Maggi G, Quinteros Hinojosa F, Villagran MJ, Guasch Arevalo E, Gilsanz Rodriguez F. Renal Replacement Therapy in Acute Kidney Failure due to Rhabdomyolysis. Case Rep Crit Care. 2012;2012:603849 Epub 2012/01/01. 10.1155/2012/603849 . 24826338 PMC4010012

[58] El-Abdellati E, Eyselbergs M, Sirimsi H, Hoof VV, Wouters K, Verbrugghe W, et al An observational study on rhabdomyolysis in the intensive care unit. Exploring its risk factors and main complication: acute kidney injury. Ann Intensive Care. 2013;3(1):8 Epub 2013/03/19. 10.1186/2110-5820-3-8 . 23497406 PMC3614462

[59] Meng M, Klingensmith NJ, Coopersmith CM. New insights into the gut as the driver of critical illness and organ failure. Curr Opin Crit Care. 2017;23(2):143–8. Epub 2017/01/17. 10.1097/MCC.0000000000000386 . 28092310 PMC5373099

[60] Zhang J, Ankawi G, Sun J, Digvijay K, Yin Y, Rosner MH, et al Gut-kidney crosstalk in septic acute kidney injury. Crit Care. 2018;22(1):117 Epub 2018/05/05. 10.1186/s13054-018-2040-y . 29724256 PMC5934860

[61] Li J, Moturi KR, Wang L, Zhang K, Yu C. Gut derived-endotoxin contributes to inflammation in severe ischemic acute kidney injury. BMC Nephrol. 2019;20(1):16 Epub 2019/01/13. 10.1186/s12882-018-1199-4 . 30634931 PMC6329050

[62] Cuzick L.M. L AR, Cooper J.R. Pathophysiology of Aortic Cross-clamping In: Chiesa R., Melissano G., Zangrillo A. (eds) Thoraco-Abdominal Aorta.: Springer, Milano; 2011.

[63] Welborn MB, Oldenburg HS, Hess PJ, Huber TS, Martin TD, Rauwerda JA, et al The relationship between visceral ischemia, proinflammatory cytokines, and organ injury in patients undergoing thoracoabdominal aortic aneurysm repair. Crit Care Med. 2000;28(9):3191–7. Epub 2000/09/29. 10.1097/00003246-200009000-00013 . 11008981

[64] Peoc’h K, Nuzzo A, Guedj K, Paugam C, Corcos O. Diagnosis biomarkers in acute intestinal ischemic injury: so close, yet so far. Clin Chem Lab Med. 2018;56(3):373–85. Epub 2017/08/26. 10.1515/cclm-2017-0291 . 28841570

[65] Deftereos S, Angelidis C, Bouras G, Raisakis K, Gerckens U, Cleman MW, et al Innate immune inflammatory response in the acutely ischemic myocardium. Med Chem. 2014;10(7):653–62. 10.2174/1573406410666140806103651 . 25102201

