# Supplementary material for: Distal organ inflammation and injury after resuscitative endovascular balloon occlusion of the aorta in a porcine model of severe hemorrhagic shock
Source: PLoS One. 2020 Nov 17;15(11):e0242450. doi: 10.1371/journal.pone.0242450 (PMC7671515; doi:10.1371/journal.pone.0242450)
Supplement: S1 Table — (DOCX) [file pone.0242450.s001.docx]

**Distal organ inflammation and injury after resuscitative endovascular balloon occlusion of the aorta in a porcine model of severe hemorrhagic shock**

Yansong Li*^1^, Michael A. Dubick^2^, Zhangsheng Yang^1^, Johnny L. Barr^2^, Brandon J. Gremmer^1^, Michael L. Lucas^1^, Corina Necsoiu^1^, Bryan S. Jordan^1^, Andriy I. Batchinsky^1^, and Leopoldo C. Cancio^3^

**Supplemental Data**

**S1 Table. Vital signs of individual animals**

| **Parameters** | **Group** | **Animal** | **Timeline** | | | | | |
| --- | --- | --- | --- | --- | --- | --- | --- | --- |
|  |  |  | **BL** | **EH** | **R90** | **R150** | **R210** | **EOS** |
| **SBP (mmHg)** | **PC** | **1** | 165 | 40 | 69 | n/a | n/a | n/a |
|  |  | **2** | 122 | 36 | 118 | 154 | 140 | 150 |
|  |  | **3** | 127 | 94 | 135 | 123 | 117 | 119 |
|  |  | **4** | 150 | 47 | 156 | 154 | 163 | 151 |
|  |  | **5** | 137 | 54 | n/a | n/a | n/a | 14 |
|  |  | **6** | 170 | 47 | 143 | 163 | n/a | 168 |
|  |  | **7** | 115 | 47 | 114 | 113 | 111 | 124 |
|  | **A30** | **1** | 129 | 57 | 139 | 161 | 179 | 185 |
|  |  | **2** | 168 | 73 | 147 | 201 | 209 | 210 |
|  |  | **3** | 115 | 57 | 113 | 134 | 155 | 162 |
|  |  | **4** | 139 | 61 | 124 | 151 | 162 | 164 |
|  |  | **5** | 124 | 65 | 135 | 194 | 175 | 134 |
|  |  | **6** | 165 | 63 | 159 | 214 | 231 | 224 |
|  |  | **7** | 134 | 62 | 125 | 172 | 184 | 183 |
|  | **A60** | **1** | 129 | 62 | 132 | 125 | 138 | 136 |
|  |  | **2** | 124 | 37 | 118 | 100 | 93 | 100 |
|  |  | **3** | 132 | 43 | 120 | 114 | 126 | 133 |
|  |  | **4** | 136 | 56 | 99 | 83 | 106 | 111 |
|  |  | **5** | 134 | 71 | 97 | 100 | 110 | 113 |
|  |  | **6** | 130 | 79 | 99 | 120 | 117 | 115 |
|  |  | **7** | 139 | 63 | 123 | 126 | 142 | 148 |
| **DBP (mmHg)** | **PC** | **1** | 135 | 13 | 29 | n/a | n/a | n/a |
|  |  | **2** | 97 | 15 | 87 | 120 | 108 | 108 |
|  |  | **3** | 90 | 96 | 103 | 84 | 85 | 85 |
|  |  | **4** | 109 | 24 | 120 | 109 | 115 | 106 |
|  |  | **5** | 99 | 16 | n/a | n/a | n/a | 13 |
|  |  | **6** | 125 | 20 | 108 | 112 | n/a | 113 |
|  |  | **7** | 68 | 22 | 70 | 69 | 68 | 78 |
|  | **A30** | **1** | 83 | 41 | 100 | 112 | 126 | 130 |
|  |  | **2** | 118 | 46 | 104 | 153 | 162 | 162 |
|  |  | **3** | 80 | 20 | 77 | 90 | 112 | 120 |
|  |  | **4** | 103 | 40 | 89 | 113 | 127 | 128 |
|  |  | **5** | 65 | 41 | 84 | 123 | 120 | 91 |
|  |  | **6** | 125 | 30 | 109 | 151 | 165 | 158 |
|  |  | **7** | 110 | 43 | 89 | 131 | 139 | 129 |
|  | **A60** | **1** | 89 | 53 | 93 | 83 | 97 | 93 |
|  |  | **2** | 81 | 27 | 63 | 56 | 50 | 53 |
|  |  | **3** | 92 | 28 | 81 | 85 | 95 | 102 |
|  |  | **4** | 101 | 26 | 52 | 42 | 62 | 66 |
|  |  | **5** | 101 | 46 | 65 | 68 | 75 | 72 |
|  |  | **6** | 102 | 44 | 59 | 72 | 69 | 66 |
|  |  | **7** | 110 | 43 | 83 | 80 | 100 | 101 |
| **MAP (mmHg)** | **PC** | **1** | 154 | 20 | 41 | n/a | n/a | n/a |
|  |  | **2** | 112 | 19 | 103 | 133 | 127 | 129 |
|  |  | **3** | 110 | 60 | 121 | 106 | 104 | 105 |
|  |  | **4** | 129 | 32 | 143 | 127 | 138 | 124 |
|  |  | **5** | 119 | 27 | n/a | n/a | n/a | 13 |
|  |  | **6** | 145 | 29 | 125 | 134 | n/a | 138 |
|  |  | **7** | 92 | 28 | 93 | 92 | 91 | 100 |
|  | **A30** | **1** | 108 | 31 | 122 | 135 | 151 | 155 |
|  |  | **2** | 140 | 57 | 128 | 174 | 183 | 182 |
|  |  | **3** | 97 | 37 | 94 | 112 | 132 | 140 |
|  |  | **4** | 123 | 49 | 110 | 134 | 144 | 144 |
|  |  | **5** | 92 | 49 | 113 | 153 | 149 | 106 |
|  |  | **6** | 147 | 41 | 135 | 180 | 189 | 185 |
|  |  | **7** | 122 | 51 | 107 | 149 | 160 | 156 |
|  | **A60** | **1** | 110 | 44 | 113 | 104 | 116 | 109 |
|  |  | **2** | 103 | 31 | 87 | 73 | 67 | 72 |
|  |  | **3** | 110 | 34 | 102 | 96 | 112 | 114 |
|  |  | **4** | 121 | 37 | 69 | 55 | 80 | 85 |
|  |  | **5** | 114 | 57 | 49 | 84 | 91 | 90 |
|  |  | **6** | 115 | 54 | 76 | 92 | 89 | 84 |
|  |  | **7** | 123 | 52 | 100 | 103 | 120 | 122 |
